# MOKPE: drug–target interaction prediction via manifold optimization based kernel preserving embedding

**DOI:** 10.1186/s12859-023-05401-1

**Published:** 2023-07-05

**Authors:** Oğuz C. Binatlı, Mehmet Gönen

**Affiliations:** 1 Graduate School of Sciences and Engineering, Koç University, 34450 Istanbul, Turkey; 2 Department of Industrial Engineering, College of Engineering, Koç University, 34450 Istanbul, Turkey; 3 School of Medicine, Koç University, 34450 Istanbul, Turkey

**Keywords:** Drug–target interaction prediction, Drug repurposing, Manifold optimization, Kernel methods, Machine learning

## Abstract

**Background:**

In many applications of bioinformatics, data stem from distinct heterogeneous sources. One of the well-known examples is the identification of drug–target interactions (DTIs), which is of significant importance in drug discovery. In this paper, we propose a novel framework, manifold optimization based kernel preserving embedding (MOKPE), to efficiently solve the problem of modeling heterogeneous data. Our model projects heterogeneous drug and target data into a unified embedding space by preserving drug–target interactions and drug–drug, target–target similarities simultaneously.

**Results:**

We performed ten replications of ten-fold cross validation on four different drug–target interaction network data sets for predicting DTIs for previously unseen drugs. The classification evaluation metrics showed better or comparable performance compared to previous similarity-based state-of-the-art methods. We also evaluated MOKPE on predicting unknown DTIs of a given network. Our implementation of the proposed algorithm in R together with the scripts that replicate the reported experiments is publicly available at https://github.com/ocbinatli/mokpe.

## Background

**Fig. 1:**
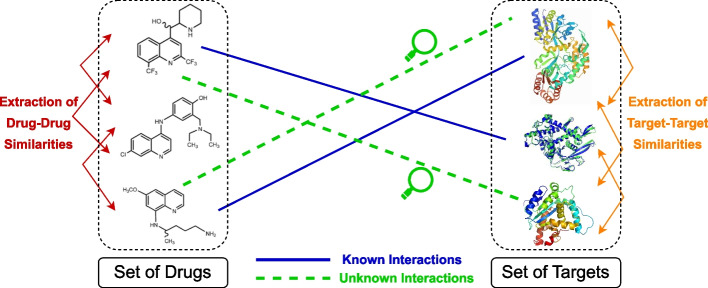
The conceptual schema of predicting DTI problem

Many applications and problems in bioinformatics require data originated from heterogeneous sources. One of the well-studied examples is the in silico identification of interactions between drugs and target proteins, which is a key area in genomic drug discovery and drug repurposing [1]. High financial costs of conducting wet lab experiments to discover new interactions leads to a strong incentive to develop computational methods capable of detecting these potential drug-target interactions (DTIs) efficiently. In DTI prediction problem, we have heterogeneous data from two domains, drugs and targets. The cross-domain interactions correspond to the given data of experimentally validated drug–target interactions. The within-domain similarity scores correspond to the chemical similarities for drug–drug networks, and genomic similarities for target–target networks. We want to approximate drug–target interactions, drug–drug and target–target similarities with Gaussian kernels to transfer local neighborhood information of the heterogeneous data to the projected subspace. Figure 1 shows the problem of predicting unknown DTIs using known DTIs and drug–drug, target–target similarities, as a conceptual illustration.

Recently, many machine learning based methods, which transform knowledge about drugs, targets and known interactions into features that are pipelined to train predictive models, have been developed. These machine learning models have been used to predict DTIs for drug repurposing or drug discovery, therefore, developing explainable and accurate novel models has gained attraction in the past decades with the advance of computational power and data analysis techniques. Recent global research effort on emerging infectious diseases (e.g., COVID-19) also shows the importance of the predictive models when the need of developing effective treatments is urgent [2, 3]. For recent comprehensive surveys on DTI prediction models, we refer the reader to [4–8].

Section “Material and methods” introduces the proposed embedding method, called *manifold optimization based kernel preserving embedding* (MOKPE), the data sets, and the experimental setup. Section “Experiments and results” explains comparison procedure against the state-of-the-art similarity-based algorithms and evaluates MOKPE over four different data sets on the task of (i) predicting DTIs for unseen drugs and (ii) predicting unknown DTIs of a given network.

## Material and methods

**Fig. 2:**
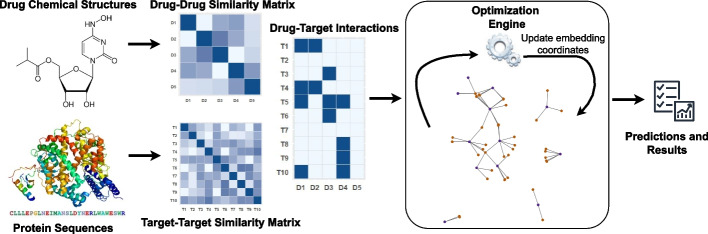
The workflow of predicting drug target interactions from the drug chemical structures and the target (protein) sequences

In this work, we follow the general framework of *multiple kernel preserving embedding* (MKPE) method, developed by Gönen [9], and propose preserving cross-domain interactions and within-domain similarities of heterogeneous data simultaneously by approximating them with kernels. Projecting the heterogeneous data into a unified embedding space is the central idea of our model formulation. To model both drug–target interactions and drug–drug, target–target similarities, we assume that these are given as scoring functions and we want to approximate these values in the projected space with kernel function values calculated in low-dimensional representations. We employ the limited-memory Riemannian BFGS method-based algorithm (LRBFGS) of [10] to solve the corresponding optimization sub-problems, which are non-convex quadratic problems with orthogonality constraints. Our framework can also be used with other cross-domain information retrieval tasks after defining scoring functions for cross-domain interactions and within-domain similarities. Figure 2 illustrates the overview of our proposed optimization framework. It should be noted that our algorithmic framework can be extended for problems with more than two domains (e.g., modeling drug–target–disease interactions) (see S1, Additional file 1 for a detailed description of MOKPE).

To evaluate the performance of our algorithm, we tested it on the task of modeling four different biological interaction networks and we compare it against the state-of-the-art algorithms based on different type of techniques. We used gold standard drug–target interaction data sets provided by [11]. We implement our algorithm in R programming language (version 4.0.2 [12]), and the source codes are publicly available at https://github.com/ocbinatli/mokpe/. The source codes for the other algorithms that we compared our method to are from [5, 13, 14].

All of the four data sets we used, Nuclear receptors (NR), G-protein-coupled receptors (GPCR), Ion channel (IC), and Enzyme (E), are important target families and publicly available at http://web.kuicr.kyoto-u.ac.jp/supp/yoshi/drugtarget/ [11]. The drug–drug and target–target similarity matrices are composed of KEGG LIGAND and KEGG GENES databases, respectively [15]. The adjacency matrices are composed of the interaction information provided by KEGG BRITE [15], BRENDA [16], SuperTarget [17], and DrugBank [18] databases [11]. Table 1 provides important information for the data sets in terms of numbers of drugs, targets, and experimentally validated interactions. Sparsity levels show the imbalance between known and unknown or non-existing DTIs, which reveal the importance of extracting intrinsic information from the drug and target spaces.

**Table 1:** The drug–target interaction data sets provided by [11]

Data set	Number of drugs	Number of targets	Number of known interactions	Sparsity (%)
NR	54	26	90	93.59
GPCR	223	95	635	97.00
IC	210	204	1476	96.55
Enzyme	445	664	2926	99.01

We formulate the problem of modeling drug–target interaction networks as follows: $\mathcal {D}$ and $\mathcal {T}$ correspond to sets of drugs and targets, respectively. The cross-domain interactions, namely, the set of experimentally validated drug–target interactions, are usually represented in the form of a binary matrix (i.e., 1 for the interacting pairs and 0 for the non-interacting (unknown) pairs). We construct our cross-domain interaction score from this binary interaction matrix as follows:

$$\begin{aligned} s_{\texttt {c},j}^{i} = {\left\{ \begin{array}{ll} 0.9 & \text {if }\varvec{d}_{i}\text { and }\varvec{t}_{j}\text { are interacting,} \\ \texttt {NA} & \text {otherwise.} \end{array}\right. } \end{aligned}$$

We set the interaction score to 0.9 for the interacting pairs. We leave the interaction score empty for the non-interacting pairs.

The chemical similarity score between two drug compounds is found by representing them as graphs and the Jaccard similarity coefficient is calculated over the substructures of these two graphs [19]. Given two drugs $\varvec{d}_{i}$ and $\varvec{d}_{j}$, chemical similarity score between them can be found as follows:

$$\begin{aligned} s_{\texttt {d},j}^{i} = \dfrac{|\varvec{d}_{i} \cap \varvec{d}_{j}|}{|\varvec{d}_{i} \cup \varvec{d}_{j}|}. \end{aligned}$$

The sequence similarity score between targets is found using a normalized version of Smith-Waterman score [20]. Given two targets $\varvec{t}_{i}$ and $\varvec{t}_{j}$, genomic similarity score between them can be found as follows:

$$\begin{aligned} s_{\texttt {t},j}^{i} = \dfrac{\texttt {SW}(\varvec{t}_{i}, \varvec{t}_{j})}{\sqrt{\texttt {SW}(\varvec{t}_{i}, \varvec{t}_{i}) \texttt {SW}(\varvec{t}_{j}, \varvec{t}_{j})}}, \end{aligned}$$

where $\texttt {SW}(\cdot , \cdot )$ gives the canonical Smith-Waterman score between two proteins. We use Gaussian kernels to approximate both similarity scores.

Our algorithm requires solving many non-convex quadratic optimization sub-problems with orthogonality constraints. Broyden-Fletcher-Goldfarb-Shanno algorithm (BFGS) is a commonly used iterative method for solving non-convex unconstrained optimization problems. It is a quasi-newton method that require only the gradient of the objective function to be supplied at each iteration, and measures the gradient vector differences to approximate the inverse of the Hessian [21]. Limited-memory BFGS (L-BFGS) is a computationally more efficient variant of BFGS, which stores and uses only the most recent solutions and gradient vectors to approximate the inverse Hessian [22, Chapter 9]. L-BFGS is widely used in non-convex unconstrained optimization and is known to perform well against the competitors (e.g., stochastic gradient descent, conjugate gradient), especially for low dimensional problems [21, 23]. Both BFGS and L-BFGS have extensions to Riemannian manifolds [10, 24, 25] which are suitable for our problem on optimization over the Stiefel manifold. We use *limited-memory Riemannian BFGS* (LRBFGS) of [10] which performed best in our preliminary experiments whereas Riemannian BFGS (RBFGS) of [10] follows closely. Other benchmarks demonstrate similar results [10, 26, 27]. In the preliminary experiments, we also employed conjugate gradient, or stochastic gradient based manifold optimization algorithms [28, 29], and they yielded only slightly better or similar results compared to our baseline algorithm, MKPE, steepest descent with Armijo-type line search (see S2, Additional file 1 for a detailed comparison of MOKPE and MKPE).

## Experiments and results

Main computational bottleneck in solving our proposed model is the complexity of optimization on the Stiefel manifold. In [9], a steepest descent method with Armijo’s rule based line search procedure was used. However, the batch steepest descent method may have slow rate of convergence [21, Chapter 3]. Therefore, we propose to use the algorithms specifically tailored to solve the optimization problems on Stiefel manifold. Throughout the last decade, many manifold optimization libraries have been released for use in different programming languages and machine learning frameworks (e.g., C++ [26], Julia [30, 31], Matlab [32], Python [33, 34], PyTorch [35, 36], R [37, 38], Tensorflow [39]). Since the manifold optimization libraries and accompanying Riemannian optimization algorithms are still-evolving, promising lines of research [40, 41], using a manifold optimization library for our problem will provide a flexible framework which is easy to modify for novel algorithms, and will result with possible future improvements in terms of both evaluation metrics and computation times.

We show the performance of our out-of-sample embedding in predicting interactions for unseen drugs. For all four data sets, we conduct ten replications of ten-fold cross-validation to test our model over previously unseen drugs. Drugs of training set are not included in the testing set. In this work, we employ ManifoldOptim  (version 1.0.1 [38]), which is an R wrapper to C++ manifold optimization library ROPTLIB [26], for employing the algorithm LRBFGS in R. We use the default stopping criterion with default values to solve the sub-problems for NR, GPCR, and IC data sets. Due to the large size of the Enzyme data set, high computational time is needed, and we set the value of the stopping criteria tolerance to $10^{-4}$ when solving the sub-problems over the manifolds of drugs and targets. We use the default values for all other input parameters. We perform 25 iterations for all data sets since the training process usually converges between 15-25 iterations before the model starts overfitting in terms of AUROC values. For relatively smaller data sets NR and GPCR, the subspace dimensionality parameter, *R*, is set to 25, which is taken from $\{5,10,15,20,25\}$. For IC and Enzyme data sets, *R* is set to be 10, and 15, respectively, which is taken from $\{5,10,15\}$. In general, we see an increasing trend in performance measures for predicting DTIs with increasing subspace dimensionality, which is theoretically expected. It is possible to improve the results for all data sets (except NR, due to its small size) by adding more dimensions to the common subspace. MOKPE starts from randomly chosen points on both Stiefel manifolds and we use QR decomposition when randomly projecting matrices onto these manifolds during the initialization step [42].

We compare our method with some baseline and state-of-the-art algorithms, that utilize different types of techniques (e.g., neighborhood methods, matrix factorization, graph-based, bipartite local models) and that are among the highest performing methods in their respective categories. In nearest profile method (NP), the interaction profile of an unseen drug is calculated via its chemically most similar nearest neighbor’s interaction profile [11]. The weighted profile method (WP) is a generalized version of NP, instead of the nearest compound, a weighted average of the unseen drug’s similarities and their interaction profiles are used [11]. Laplacian Regularized Least Squares (LapRLS) is a semi-supervised learning framework that uses both labeled and unlabeled DTIs, and obtains predictions from both drug and target sides and combines them [43]. Its objective function contains the minimization of prediction error and also includes a manifold regularization term that extends regularized least squares (RLS) with a Laplacian operator. While LapRLS estimates the drug and target spaces separately, Dual Laplacian Regularized Least Squares (DLapRLS) [13] approximates the interaction matrix of DTIs with interdependence of two spaces, by employing alternating least squares algorithm to solve the model, and the dual Laplacian regularization is used to smooth the weights. RLS-WNN is another RLS-based method that incorporates products of Gaussian kernels (GIP) constructed from DTI profiles [44]. A pre-processing algorithm is used to approximate the drug interaction score profile for unseen drugs using the weighted nearest neighbors (WNN), and it is combined with GIP. Kernelized Bayesian matrix factorization with twin kernels (KBMF2K)[45], collaborative matrix factorization (CMF) [46], weighted graph regularized matrix factorization (WGRMF) [47], and graph regularized generalized matrix factorization (GRGMF) [14] are the methods that approximate the DTI matrix by matrix decomposition. KBMF2K employs a Bayesian formulation and uses variational approximation to project drugs and proteins into a unified subspace. CMF uses collaborative filtering, jointly approximating DTI matrix via two low-rank matrices that share the same subspace and approximate drug–drug and target–target similarity matrices. WGRMF is similar to CMF, but it preprocesses the interaction matrix to transform the binary values into interaction values with weighted nearest neighbor algorithm, and uses graph regularization for manifold learning to approximate the similarity matrices. GRGMF presents a model for predicting links in bipartite networks. The model is based on the assumption that the latent factor of each node, which is learned adaptively by its neighborhood information, of drugs and targets are correlated with each other and that the correlation can be represented by a bipartite graph. Heterogeneous graph based inference (HGBI) [48] is an extended version of network-based inference method [49] that uses drug-target bipartite graph network similarity. Instead of a bipartite network, HGBI uses a network diffusion with incorporating drug–drug and target–target similarities on a heterogeneous network. For both MOKPE and compared methods, same testing and training drug sets are used in the experiments. Drugs in the test set are not present in the training set. In MOKPE, they are excluded both from the interaction matrix and the similarity matrices. Reported best hyper-parameters taken from [5, 13, 14] are used when running the compared methods (see S3, Additional file 1 for a detailed description of parameters for all methods).

**Table 2:** Average AUROC results (and standard deviations)

Methods	Data set			
	NR	GPCR	IC	E
Nearest profile	0.762* (0.013)	0.771* (0.004)	0.623* (0.010)	0.715* (0.012)
Weighted profile	0.768* (0.016)	0.813* (0.005)	0.765* (0.007)	0.783* (0.013)
LapRLS	0.760* (0.019)	0.810* (0.005)	0.752* (0.007)	0.774*(0.013)
DLapRLS	0.826 (0.015)	0.807* (0.011)	0.755* (0.012)	0.754* (0.011)
RLS-WNN	0.856 (0.015)	0.870 (0.006)	0.808 (0.009)	0.800* (0.012)
KBMF2K	0.798* (0.012)	0.810* (0.009)	0.792 (0.009)	0.724* (0.013)
CMF	0.806* (0.018)	0.807* (0.009)	0.767* (0.011)	0.795* (0.010)
WGRMF	0.874 (0.013)	0.878 (0.006)	0.801 (0.009)	0.822 (0.009)
GRGMF	**0.874 (0.011)**	**0.879 (0.005)**	**0.814 (0.009)**	**0.825 (0.015)**
HGBI	0.777* (0.013)	0.813* (0.004)	0.718* (0.008)	0.809 (0.014)
MOKPE	0.850 (0.015)	0.878 (0.005)	0.800 (0.007)	0.824 (0.010)

* indicates that MOKPE significantly outperforms this method with $p < 0.05$ using Mann–Whitney test. The highest result in each column is bolded and the second best is underlined

**Table 3:** Average AUPRC results (and standard deviations)

Methods	Data set			
	NR	GPCR	IC	E
Nearest Profile	0.427* (0.017)	0.275* (0.017)	0.209* (0.014)	0.225* (0.023)
Weighted Profile	0.380* (0.022)	0.231* (0.006)	0.193* (0.007)	0.116* (0.003)
LapRLS	0.367* (0.026)	0.219* (0.006)	0.177* (0.007)	0.110* (0.002)
DLapRLS	0.415* (0.026)	0.386 (0.008)	0.320 (0.017)	0.365 (0.018)
RLS-WNN	0.558 (0.023)	0.369 (0.012)	0.342 (0.019)	0.380 (0.022)
KBMF2K	0.491* (0.020)	0.380 (0.010)	0.316 (0.016)	0.249 (0.017)
CMF	0.528* (0.019)	0.400 (0.008)	0.355 (0.018)	0.391 (0.016)
WGRMF	**0.592 (0.018)**	**0.420 (0.011)**	**0.378 (0.022)**	**0.409 (0.019)**
GRGMF	0.506* (0.015)	0.367 (0.009)	0.366 (0.023)	0.363 (0.026)
HGBI	0.275* (0.024)	0.204* (0.011)	0.115* (0.009)	0.107* (0.006)
MOKPE	0.578 (0.023)	0.374 (0.008)	0.321 (0.015)	0.244 (0.016)

* indicates that MOKPE significantly outperforms this method with $p < 0.05$ using Mann–Whitney test. The highest result in each column is bolded and the second best is underlined

**Fig. 3:**
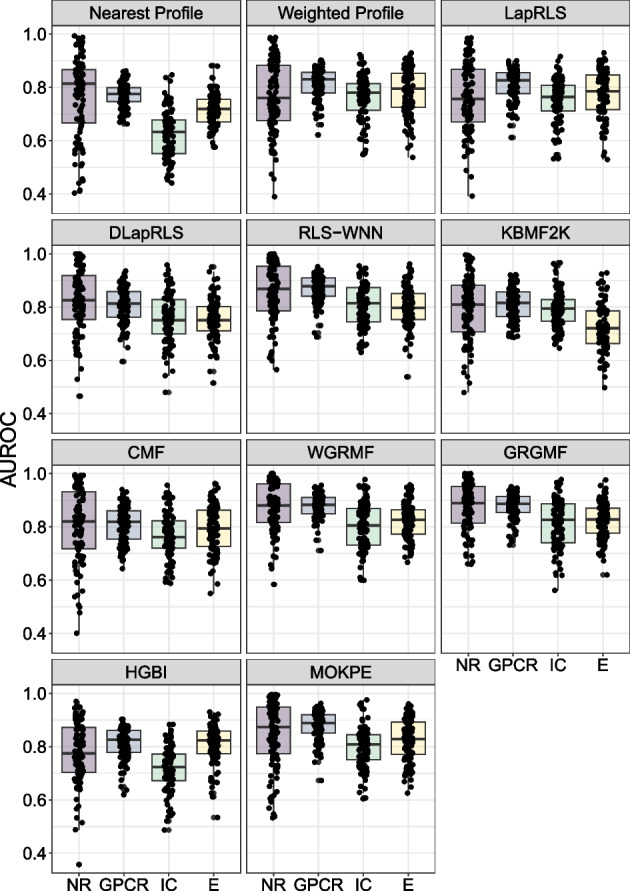
Boxplots to illustrate the prediction performance of MOKPE and compared algorithms on the NR, GPCR, IC and E data sets. Each point shows the evaluation of a test set in terms of AUROC value

**Fig. 4:**
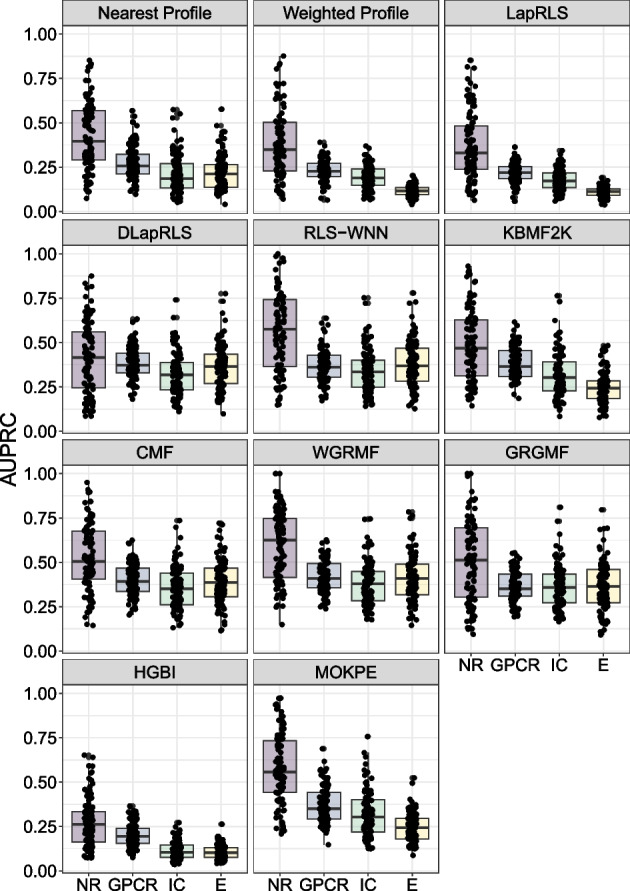
Boxplots to illustrate the prediction performance of MOKPE and compared algorithms on the NR, GPCR, IC and E data sets. Each point shows the evaluation of a test set in terms of AUPRC value

**Fig. 5:**
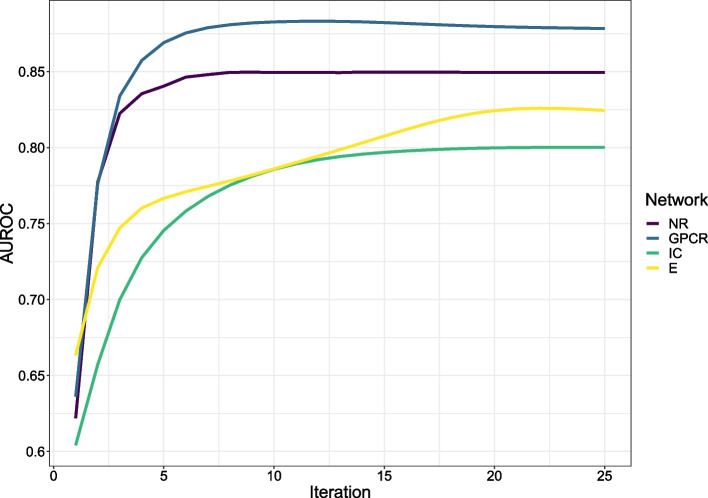
The classification performance of MOKPE with increasing number of iterations in terms of average AUROC values

**Fig. 6:**
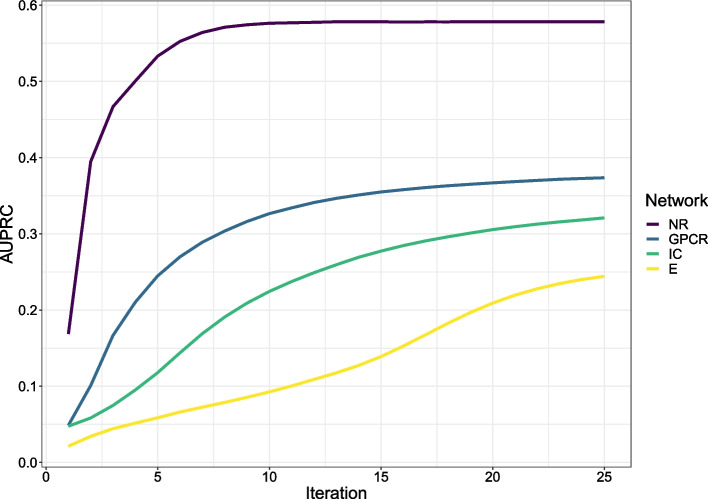
The classification performance of MOKPE with increasing number of iterations in terms of average AUPRC values

Tables 2 and 3 give the average AUROC (area under the receiver operating characteristic curve) and AUPRC (area under the precision-recall curve) values for MOKPE and compared algorithms. Best and the second best results in each column are bolded and underlined, respectively. In Figs. 3 and 4, we see the AUROC and AUPRC values for MOKPE and compared algorithms for each test set in the boxplots. Figs. 5 and 6 give the average AUROC and AUPRC values for MOKPE with changing number of iterations. For MOKPE, we want to emphasize that there is an increasing trend for results with increasing subspace dimensionality for all data sets. It is anticipated that the prediction performances in terms of AUPRC values might be improved on IC and Enzyme data sets by increasing the number of iterations, increasing the subspace dimensionality and by fully exploiting the hyper-parameter space, especially for the stopping criteria of the sub-problems. Our experiments show that the results on the NR data set are unstable likely due to its small size, as aligned with previous research [5, 47, 50]. For the IC data set, all methods perform poorly compared to other data sets. Although the IC data set has more targets and more known interactions compared to the GPCR data set, the lesser ratio of the number of drugs to the number of targets may explain the phenomena [6]. Moreover, since the information for drugs is more valuable for the IC data set, this may give a disadvantage for methods (e.g., MOKPE) that have empty profiles for drug–drug similarities for unseen drugs, and may explain the better results for methods that use the drug–drug similarity information for tested drugs (e.g., RLS-WNN with its preprocessing for constructing temporary interaction profiles for unseen drugs). We note that many methods (e.g., DLapRLS, WGRMF, GRGMF) utilize preprocessed known interactions via WKNKN (weighted k-nearest known neighbors) algorithm to estimate the associations for the unseen drugs using drug–drug similarity matrix and improve their performance. We also note that RLS-WNN is a faster algorithm among the best performing algorithms. Matrix factorization methods are slower, although they are relatively better in predicting DTIs. MOKPE is slower against its competitors, however, the implementation of the objective and gradient functions in C++ would yield with a faster performance as the ManifoldOptim developers noted [38]. The possible effects of newer algorithm developments, performance and quality improvements in manifold optimization algorithms will also be seen easily in our framework.

### Validation of newly predicted drug–target interactions

**Fig. 7:**
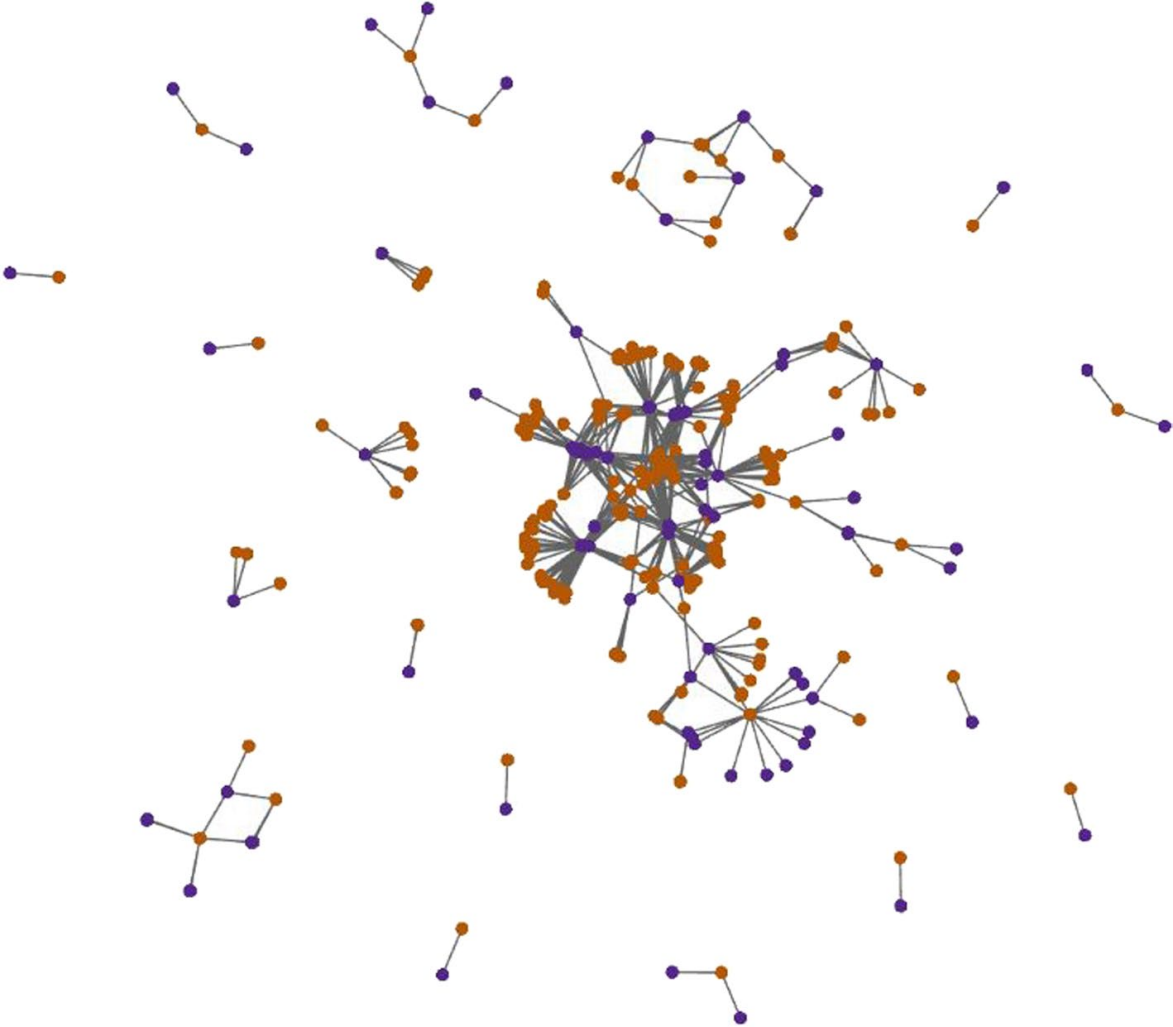
The two-dimensional embeddings on the GPCR data set. Orange and purple points denote drugs and targets, respectively

To show the effectiveness of our method, we also look for the prediction of new DTIs that are unknown in the original data sets. The data sets we used [11] were developed over a decade ago, and many novel DTIs were discovered after the compilation of original data sets. In these second set of experiments, we use the entire data set as a training set, and project drugs and proteins into a two-dimensional embedding space (i.e. subspace dimensionality *R* is equal to 2) for the data sets NR and GPCR, ten-dimensional and fifty-dimensional embedding spaces for the data sets IC and Enzyme, respectively. The algorithm is terminated when the improvement over the objective function value of the training loss is smaller than $10^{-6}$ for two consecutive outer iterations. For the inner iterations, the default parameter values are used for all data sets when calling the manifold optimization library. We rank the novel predictions according to their Euclidean distances in the embedding space and list the top twenty-five ranked interactions for the above mentioned data sets. We check the novel interactions using updated curated databases KEGG [15, 51, 52], DrugBank [53], Comparative Toxicogenomics Database (CTD) [54], Guide to Pharmacology (GtP) [55], the Drug-Gene Interaction Database (DGIdb) [56], and Drug Target Commons (DTC) [57] to validate our results. Tables 4, 5, 6 and 7 lists the top twenty-five interactions for NR, GPCR, IC, and Enzyme data sets, and it can be seen that 27 out of 100 novel DTIs are validated by the sources. It should be noted that the invalidations for DTIs are rarely reported [58], and the absence of a validation does not necessarily mean a false positive. We also illustrate and publish two-dimensional embeddings for all four data sets (e.g., Fig. 7), along with the corresponding embedding coordinates and the top twenty-five ranked predictions, which can be seen and downloaded at https://ocbinatli.shinyapps.io/embedding_networks.

**Table 4:** Top-25 ranked novel DTIs predicted on the NR data set by MOKPE

Drug ID	Drug name	Target ID	Target name	Validation source
Data Set: NR (*R*=2)				
D00462	Oxandrolone	hsa4306	NR3C2 (Nuclear Receptor Subfamily 3 Group C Member 2)	None
D00348	Isotretinoin	hsa6096	RORB (RAR Related Orphan Receptor B)	None
D00690	Mometasone furoate	hsa2908	NR3C1 (Nuclear Receptor Subfamily 3 Group C Member 1)	KEGG & DRUGBANK
D00075	Testosterone	hsa5241	PGR (Progesterone Receptor)	CTD
D00088	Hydrocortisone	hsa5241	PGR (Progesterone Receptor)	None
D00962	Clomiphene citrate	hsa2101	ESRRA (Estrogen Related Receptor Alpha)	None
D00898	Dienestrol	hsa2101	ESRRA (Estrogen Related Receptor Alpha)	None
D00348	Isotretinoin	hsa5915	RARB (Retinoic Acid Receptor Beta)	KEGG & CTD
D00956	Nandrolone phenpropionate	hsa4306	NR3C2 (Nuclear Receptor Subfamily 3 Group C Member 2)	None
D00348	Isotretinoin	hsa5916	RARG (Retinoic Acid Receptor Gamma)	KEGG & DRUGBANK & CTD
D00443	Spironolactone	hsa2908	NR3C1 (Nuclear Receptor Subfamily 3 Group C Member 1)	DRUGBANK
D00348	Isotretinoin	hsa6097	RORC (RAR Related Orphan Receptor C)	None
D00956	Nandrolone phenpropionate	hsa5241	PGR (Progesterone Receptor)	None
D00956	Nandrolone phenpropionate	hsa2908	NR3C1 (Nuclear Receptor Subfamily 3 Group C Member 1)	None
D00962	Clomiphene citrate	hsa2104	ESRRG (Estrogen Related Receptor Gamma)	None
D00898	Dienestrol	hsa2104	ESRRG (Estrogen Related Receptor Gamma)	None
D00962	Clomiphene citrate	hsa2103	ESRRB (Estrogen Related Receptor Beta)	None
D00316	Etretinate	hsa6096	RORB (RAR Related Orphan Receptor B)	None
D00962	Clomiphene citrate	hsa2100	ESR2 (Estrogen Receptor 2)	KEGG
D00951	Medroxyprogesterone acetate	hsa2908	NR3C1 (Nuclear Receptor Subfamily 3 Group C Member 1)	CTD
D00898	Dienestrol	hsa2103	ESRRB (Estrogen Related Receptor Beta)	None
D00898	Dienestrol	hsa2100	ESR2 (Estrogen Receptor 2)	KEGG
D00075	Testosterone	hsa2908	NR3C1 (Nuclear Receptor Subfamily 3 Group C Member 1)	DTC & CTD
D00075	Testosterone	hsa4306	NR3C2 (Nuclear Receptor Subfamily 3 Group C Member 2)	DRUGBANK & CTD
D00327	Fluoxymesterone	hsa5241	PGR (Progesterone Receptor)	None

**Table 5:** Top-25 ranked novel DTIs predicted on the GPCR data set by MOKPE

Drug ID	Drug name	Target ID	Target name	Validation source
Data Set: GPCR (*R*=2)				
D01692	Alfuzosin hydrochloride	hsa152	ADRA2C (Adrenoceptor Alpha 2C)	None
D00613	Fenoldopam mesylate	hsa11255	HRH3 (Histamine Receptor H3)	None
D00613	Fenoldopam mesylate	hsa59340	HRH4 (Histamine Receptor H4)	None
D00965	Nilutamide	hsa152	ADRA2C (Adrenoceptor Alpha 2C)	None
D04375	Guanabenz	hsa152	ADRA2C (Adrenoceptor Alpha 2C)	KEGG & GtP
D00318	Famotidine	hsa152	ADRA2C (Adrenoceptor Alpha 2C)	None
D00380	Tolbutamide	hsa134	ADORA1 (Adenosine A1 Receptor)	None
D00514	Dexmedetomidine	hsa152	ADRA2C (Adrenoceptor Alpha 2C)	KEGG & GtP
D02327	Doxylamine succinate	hsa1131	CHRM3 (Cholinergic Receptor Muscarinic 3)	None
D02234	Cyproheptadine hydrochloride	hsa11255	HRH3 (Histamine Receptor H3)	None
D02327	Doxylamine succinate	hsa1133	CHRM5 (Cholinergic Receptor Muscarinic 5)	None
D02340	Loxapine	hsa1129	CHRM2 (Cholinergic Receptor Muscarinic 2)	DRUGBANK
D01965	Silodosin	hsa152	ADRA2C (Adrenoceptor Alpha 2C)	None
D01973	Eletriptan hydrobromide	hsa3356	HTR2A (5-Hydroxytryptamine Receptor 2A)	None
D00079	Dinoprostone	hsa5731	PTGER1 (Prostaglandin E Receptor 1)	DRUGBANK & DTC & CTD & GtP
D02357	Methysergide	hsa3360	HTR4 (5-Hydroxytryptamine Receptor 4)	None
D02340	Loxapine	hsa1128	CHRM1 (Cholinergic Receptor Muscarinic 1)	DRUGBANK
D02349	Dipivefrin	hsa3274	HRH2 (Histamine Receptor H2)	None
D02327	Doxylamine succinate	hsa1132	CHRM4 (Cholinergic Receptor Muscarinic 4)	None
D01020	Methoxamine hydrochloride	hsa152	ADRA2C (Adrenoceptor Alpha 2C)	None
D00437	Nifedipine	hsa3274	HRH2 (Histamine Receptor H2)	None
D02349	Dipivefrin	hsa152	ADRA2C (Adrenoceptor Alpha 2C)	KEGG
D02327	Doxylamine succinate	hsa1814	DRD3 (Dopamine Receptor D3)	None
D01164	Aripiprazole	hsa1129	CHRM2 (Cholinergic Receptor Muscarinic 2)	DRUGBANK
D01297	Pirenzepine hydrochloride	hsa1131	CHRM3 (Cholinergic Receptor Muscarinic 3)	KEGG

**Table 6:** Top-25 ranked novel DTIs predicted on the IC data set by MOKPE

Drug ID	Drug name	Target ID	Target name	Validation source
Data Set: IC (*R*=10)				
D00349	Isradipine	hsa773	CACNA1A (Calcium Voltage-Gated Channel Subunit Alpha1 A)	None
D00349	Isradipine	hsa777	CACNA1E (Calcium Voltage-Gated Channel Subunit Alpha1 E)	CTD
D00638	Flecainide acetate	hsa8645	KCNK5 (Potassium Two Pore Domain Channel Subfamily K Member 5)	None
D00349	Isradipine	hsa774	CACNA1B (Calcium Voltage-Gated Channel Subunit Alpha1 B)	None
D03991	Encainide hydrochloride	hsa8645	KCNK5 (Potassium Two Pore Domain Channel Subfamily K Member 5)	None
D00349	Isradipine	hsa5310	PKD1 (Polycystin 1, Transient Receptor Potential Channel Interacting)	None
D00438	Nimodipine	hsa773	CACNA1A (Calcium Voltage-Gated Channel Subunit Alpha1 A)	None
D00619	Verapamil hydrochloride	hsa3739	KCNA4 (Potassium Voltage-Gated Channel Subfamily A Member 4)	None
D03365	Nicotine	hsa1137	CHRNA4 (Cholinergic Receptor Nicotinic Alpha 4 Subunit)	KEGG & DRUGBANK & DTC & GtP
D00438	Nimodipine	hsa781	CACNA2D1 (Calcium Voltage-Gated Channel Auxiliary Subunit Alpha2delta 1)	None
D00349	Isradipine	hsa782	CACNB1 (Calcium Voltage-Gated Channel Auxiliary Subunit Beta 1)	None
D00438	Nimodipine	hsa779	CACNA1S (Calcium Voltage-Gated Channel Subunit Alpha1 S)	KEGG & DRUGBANK & GtP
D05024	Mibefradil dihydrochloride	hsa5310	PKD1 (Polycystin 1, Transient Receptor Potential Channel Interacting)	None
D05024	Mibefradil dihydrochloride	hsa774	CACNA1B (Calcium Voltage-Gated Channel Subunit Alpha1 B)	None
D00647	Dofetilide	hsa9424	KCNK6 (Potassium Two Pore Domain Channel Subfamily K Member 6)	None
D00364	Loratadine	hsa3737	KCNA2 (Potassium Voltage-Gated Channel Subfamily A Member 2)	None
D00615	Amlodipine besylate	hsa774	CACNA1B (Calcium Voltage-Gated Channel Subunit Alpha1 B)	None
D00640	Propafenone hydrochloride	hsa3743	KCNA7 (Potassium Voltage-Gated Channel Subfamily A Member 7)	None
D00619	Verapamil hydrochloride	hsa3741	KCNA5 (Potassium Voltage-Gated Channel Subfamily A Member 5)	None
D00616	Diltiazem hydrochloride	hsa3739	KCNA4 (Potassium Voltage-Gated Channel Subfamily A Member 4)	None
D01969	Gallopamil hydrochloride	hsa778	CACNA1F (Calcium Voltage-Gated Channel Subunit Alpha1 F)	KEGG
D00364	Loratadine	hsa3746	KCNC1 (Potassium Voltage-Gated Channel Subfamily C Member 1)	None
D00619	Verapamil hydrochloride	hsa3746	KCNC1 (Potassium Voltage-Gated Channel Subfamily C Member 1)	None
D00647	Dofetilide	hsa3756	KCNH1 (Potassium Voltage-Gated Channel Subfamily H Member 1)	GtP
D00619	Verapamil hydrochloride	hsa3737	KCNA2 (Potassium Voltage-Gated Channel Subfamily A Member 2)	None

**Table 7:** Top-25 ranked novel DTIs predicted on the Enzyme data set by MOKPE

Drug ID	Drug name	Target ID	Target name	Validation source
Data Set: E (*R*=50)				
D00542	Halothane	hsa1571	CYP2E1 (Cytochrome P450 Family 2 Subfamily E Member 1)	KEGG & DRUGBANK & CTD
D00377	Mesalamine	hsa246	ALOX15 (Arachidonate 15-Lipoxygenase)	None
D00377	Mesalamine	hsa239	ALOX12 (Arachidonate 12-Lipoxygenase, 12S Type)	None
D00377	Mesalamine	hsa242	ALOX12B (Arachidonate 12-Lipoxygenase, 12R Type)	None
D00377	Mesalamine	hsa4048	LTA4H (Leukotriene A4 Hydrolase)	None
D00377	Mesalamine	hsa247	ALOX15B (Arachidonate 15-Lipoxygenase Type B)	None
D00574	Aminoglutethimide	hsa1589	CYP21A2 (Cytochrome P450 Family 21 Subfamily A Member 2)	None
D00960	Anastrozole	hsa1586	CYP17A1 (Cytochrome P450 Family 17 Subfamily A Member 1)	None
D01425	Lopinavir	hsa1586	CYP17A1 (Cytochrome P450 Family 17 Subfamily A Member 1)	None
D00437	Nifedipine	hsa1585	CYP11B2 (Cytochrome P450 Family 11 Subfamily B Member 2)	CTD
D03778	Fadrozole hydrochloride hydrate	hsa1586	CYP17A1 (Cytochrome P450 Family 17 Subfamily A Member 1)	None
D00964	Letrozole	hsa1586	CYP17A1 (Cytochrome P450 Family 17 Subfamily A Member 1)	CTD
D02451	Fadrozole hydrochloride	hsa1586	CYP17A1 (Cytochrome P450 Family 17 Subfamily A Member 1)	None
D00139	Methoxsalen	hsa1543	CYP1A1 (Cytochrome P450 Family 1 Subfamily A Member 1)	DRUGBANK & CTD
D00691	Dyphylline	hsa5152	PDE9A (Phosphodiesterase 9A)	None
D03781	Liarozole fumarate	hsa1589	CYP21A2 (Cytochrome P450 Family 21 Subfamily A Member 2)	None
D00960	Anastrozole	hsa1589	CYP21A2 (Cytochrome P450 Family 21 Subfamily A Member 2)	None
D03781	Liarozole fumarate	hsa284541	CYP4A22 (Cytochrome P450 Family 4 Subfamily A Member 22)	None
D03784	Liarozole hydrochloride	hsa1589	CYP21A2 (Cytochrome P450 Family 21 Subfamily A Member 2)	None
D00960	Anastrozole	hsa284541	CYP4A22 (Cytochrome P450 Family 4 Subfamily A Member 22)	None
D03781	Liarozole fumarate	hsa8529	CYP4F2 (Cytochrome P450 Family 4 Subfamily F Member 2)	None
D00960	Anastrozole	hsa8529	CYP4F2 (Cytochrome P450 Family 4 Subfamily F Member 2)	None
D03781	Liarozole fumarate	hsa4051	CYP4F3 (Cytochrome P450 Family 4 Subfamily F Member 3)	None
D00964	Letrozole	hsa1589	CYP21A2 (Cytochrome P450 Family 21 Subfamily A Member 2)	None
D00960	Anastrozole	hsa4051	CYP4F3 (Cytochrome P450 Family 4 Subfamily F Member 3)	None

## Conclusions

Identifying drug–target interactions is crucial for drug development and repurposing. Therefore, predicting drug–target interactions with in silico applications have received extensive interest due to its importance in human biology. We have introduced a drug–target interaction prediction framework, called manifold optimization based kernel preserving embedding, which can also be used for modeling other types of biological interaction networks or cross-domain information retrieval tasks. Our method maps objects from different domains (i.e., drugs and targets) into a unified embedding space by preserving both cross-domain interactions and within-domain similarities, which are approximated with Gaussian kernels. Our framework is able to transfer local neighborhood information from the provided interactions and similarities, and to conduct out-of-sample embedding via using the non-linear kernels in the embedding space. Experimental results against state-of-the-art methods using AUROC and AUPRC evaluation metrics, and predicting novel DTIs that are validated with newer databases, show the success of our method.

The future direction of DTI prediction is expected to focus on improving the accuracy of predictions by incorporating more data sources (e.g., side-effects, biological functions, etc.) and developing more combined machine learning methods [59]. Our method can be extended to integrate multiple related data sources. Although most targets are proteins, recent studies show that it is also important to consider the interactions between drugs and small molecules (e.g., microRNAs, non-coding RNAs), and exploring the potential associations between these small molecules and diseases is crucial in the drug development process to improve the treatment of complex diseases [60]. Our method can also be used in this line of research (e.g., [61, 62]) for the predictions within this new class of drug-targets.

Employing a manifold optimization library for the optimization steps provides a flexible, easy-to-update framework. Another advantage of MOKPE is, it does not require complex hyper-parameter selection, therefore, it is simple to use and valuable in many real-life applications. Although our method demonstrates a significant performance for the gold standard data sets, further investigations of drug–target networks with larger sizes, or other large-size heterogeneous networks that are tangential to the DTI prediction evaluation (i.e., drug–disease networks) will be possible with further improvements in terms of computational cost.

## Supplementary Information

**Additional file 1.** Chap. S1. The detailed mathematical modeling and pseudo-code of MOKPE. Chap. S2. A comparison of MOKPE against the Steepest Descent Method. Chap. S3. The hyper-parameter settings that are used in the compared methods.

## Data Availability

Our implementation of proposed MOKPE algorithm in R programming language is publicly available at https://github.com/ocbinatli/mokpe. The data sets analyzed during this study are publicly available at http://web.kuicr.kyoto-u.ac.jp/supp/yoshi/drugtarget/.
