# Supplementary material for: MOKPE: drug–target interaction prediction via manifold optimization based kernel preserving embedding
Source: BMC Bioinformatics. 2023 Jul 5;24:276. doi: 10.1186/s12859-023-05401-1 (PMC10324162; doi:10.1186/s12859-023-05401-1)
Supplement: Supplementary file 1 — Additional file 1. Chap. S1. The detailed mathematical modeling and pseudo-code of MOKPE. Chap. S2. A comparison of MOKPE against the Steepest Descent Method. Chap. S3. The hyper-parameter settings that are used in the compared methods. [file 12859_2023_5401_MOESM1_ESM.pdf]

MOKPE: Drug–target interaction prediction via manifold optimization based kernel preserving embedding — Supplementary material —

Oğuz C. Binatlı[1] and Mehmet Gönen[2, 3]

[1]Graduate School of Science and Engineering, Koç University, 34450 İstanbul, Turkey
[2]Department of Industrial Engineering, College of Engineering, Koç University, 34450 İstanbul, Turkey
[3]School of Medicine, Koç University, 34450 İstanbul, Turkey

S1 Manifold Optimization based Kernel Preserving Embedding

Our data come from two different domains, namely, $\mathcal{D}$ and $\mathcal{T}$, and we are given two sets of objects $\mathbf{D} = \{\boldsymbol{d}_i \in \mathcal{D}\}_{i=1}^{N_\mathtt{d}}$ and $\mathbf{T} = \{\boldsymbol{t}_i \in \mathcal{T}\}_{i=1}^{N_\mathtt{t}}$, corresponding to drugs and targets, respectively. Usually, these objects have vectorial representations (i.e., $\mathcal{D}$ and $\mathcal{T}$ are Euclidean spaces) in practical applications, however, the domains in our work contain non-vectorial structures, such as protein sequence strings for targets. Aiming for having a general notation for both vectorial and non-vectorial data, we assume that the drug–target interactions and drug–drug, target–target similarities are provided with three different scoring functions:

i. $s_{\mathtt{c},j}^{i} \colon \mathcal{D} \times \mathcal{T} \to \mathbb{R}$ gives the drug–target interaction score between $\boldsymbol{d}_i$ and $\boldsymbol{t}_j$,

ii. $s_{\mathtt{d},j}^{i} \colon \mathcal{D} \times \mathcal{D} \to \mathbb{R}$ gives the drug–drug similarity score between drugs $\boldsymbol{d}_i$ and $\boldsymbol{d}_j$,

iii. $s_{\mathtt{t},j}^{i} \colon \mathcal{T} \times \mathcal{T} \to \mathbb{R}$ gives the target–target similarity score between targets $\boldsymbol{t}_i$ and $\boldsymbol{t}_j$.

Hence, we can use the within-domain similarity functions to represent the objects in a vectorial form, which is known as *empirical kernel map* [1, Chapter 2]:

$$\boldsymbol{d}_i = \begin{bmatrix} s_{\mathtt{d},1}^{i} & s_{\mathtt{d},2}^{i} & \dots & s_{\mathtt{d},N_\mathtt{d}}^{i} \end{bmatrix}^\top \qquad \forall i$$
$$\boldsymbol{t}_i = \begin{bmatrix} s_{\mathtt{t},1}^{i} & s_{\mathtt{t},2}^{i} & \dots & s_{\mathtt{t},N_\mathtt{t}}^{i} \end{bmatrix}^\top \qquad \forall i.$$

This technique allows us to introduce non-linearity into the embedding part [2, Chapter 2] even for the models where the objects have corresponding vectorial representations.

We also introduce three index sets, namely, $\mathcal{I}_\mathtt{c} = \{(i, j) \colon s_{\mathtt{c},j}^{i} \text{ is known}\}$, $\mathcal{I}_\mathtt{d} = \{(i, j) \colon s_{\mathtt{d},j}^{i} \text{ is known}\}$, and $\mathcal{I}_\mathtt{t} = \{(i, j) \colon s_{\mathtt{t},j}^{i} \text{ is known}\}$, to represent known information coming from these scoring functions.

We map objects from two different domains into a unified embedding space. The objects in $\mathbf{D}$ and $\mathbf{T}$ are converted into R-dimensional vectors of an Euclidean space, namely, $\mathbf{E}_\mathtt{d} = \{\boldsymbol{e}_{\mathtt{d},i} \in \mathbb{R}^R\}_{i=1}^{N_\mathtt{d}}$ and $\mathbf{E}_\mathtt{t} = \{\boldsymbol{e}_{\mathtt{t},i} \in \mathbb{R}^R\}_{i=1}^{N_\mathtt{t}}$. However, to be able to do out-of-sample embedding, we can assume linear projections from the input domains to the embedding domain and optimize the model with respect to the projection matrices. The embedding coordinates are formulated as

$$\boldsymbol{e}_{\mathtt{d},i} = \mathbf{V}_\mathtt{d}^\top \boldsymbol{d}_i \qquad \forall i$$

$$\boldsymbol{e}_{\mathtt{t},i} = \mathbf{V}_{\mathtt{t}}^{\top}\boldsymbol{t}_i \qquad \forall i,$$

where we assume that both of the objects have vectorial representations (i.e., $\mathcal{D} \in \mathbb{R}^{D_{\mathtt{d}}}$ and $\mathcal{T} \in \mathbb{R}^{D_{\mathtt{t}}}$, where $D_{\mathtt{d}}$ and $D_{\mathtt{t}}$ are subsets of $N_{\mathtt{d}}$ and $N_{\mathtt{t}}$, respectively).

We want to approximate the scoring functions $s^i_{\mathtt{c},j}$, $s^i_{\mathtt{d},j}$, and $s^i_{\mathtt{t},j}$ by three kernel functions, $k^i_{\mathtt{c},j} \colon \mathbb{R}^R \times \mathbb{R}^R \to \mathbb{R}$, $k^i_{\mathtt{d},j} \colon \mathbb{R}^R \times \mathbb{R}^R \to \mathbb{R}$, and $k^i_{\mathtt{t},j} \colon \mathbb{R}^R \times \mathbb{R}^R \to \mathbb{R}$. These three kernel functions in the embedding space have to be differentiable with respect to the projected coordinates for calculating the gradients required for the optimization step. We use the Gaussian kernel (also called the *radial basis function* kernel) in the linear projections from the input domains to the embedding space to capture the local neighborhood information coming from the cross-domain interactions and within-domain similarities. The kernel functions in the embedding space can be written as

$$\begin{aligned}
k^i_{\mathtt{c},j} &= \exp\left(-\frac{\|\boldsymbol{e}_{\mathtt{d},i} - \boldsymbol{e}_{\mathtt{t},j}\|_2^2}{\sigma_{\mathtt{e}}^2}\right) = \exp(\mathcal{V}^i_{\mathtt{c},j}) \qquad \forall (i,j) \\
k^i_{\mathtt{d},j} &= \exp\left(-\frac{\|\boldsymbol{e}_{\mathtt{d},i} - \boldsymbol{e}_{\mathtt{d},j}\|_2^2}{\sigma_{\mathtt{e}}^2}\right) = \exp(\mathcal{V}^i_{\mathtt{d},j}) \qquad \forall (i,j) \\
k^i_{\mathtt{t},j} &= \exp\left(-\frac{\|\boldsymbol{e}_{\mathtt{t},i} - \boldsymbol{e}_{\mathtt{t},j}\|_2^2}{\sigma_{\mathtt{e}}^2}\right) = \exp(\mathcal{V}^i_{\mathtt{t},j}) \qquad \forall (i,j),
\end{aligned}$$

where $\sigma_{\mathtt{e}} \in \mathbb{R}_{++}$ is the kernel width, and the auxiliary variables, namely, $\mathcal{V}^i_{\mathtt{c},j}$, $\mathcal{V}^i_{\mathtt{d},j}$, and $\mathcal{V}^i_{\mathtt{t},j}$, are used for simplicity.

We propose to preserve the interaction and similarity scores together using a compound loss function:

$$\mathcal{L} = \frac{\lambda_{\mathtt{c}}}{|\mathcal{I}_{\mathtt{c}}|}\sum_{\mathcal{I}_{\mathtt{c}}}(k^i_{\mathtt{c},j} - s^i_{\mathtt{c},j})^2 + \frac{\lambda_{\mathtt{d}}}{|\mathcal{I}_{\mathtt{d}}|}\sum_{\mathcal{I}_{\mathtt{d}}}(k^i_{\mathtt{d},j} - s^i_{\mathtt{d},j})^2 + \frac{\lambda_{\mathtt{t}}}{|\mathcal{I}_{\mathtt{t}}|}\sum_{\mathcal{I}_{\mathtt{t}}}(k^i_{\mathtt{t},j} - s^i_{\mathtt{t},j})^2,$$

where $|\cdot|$ gives the cardinality of the input set. We have separate mean squared error terms, and separate regularization hyperparameters, namely, $\lambda_{\mathtt{c}} \in \mathbb{R}_+$, $\lambda_{\mathtt{d}} \in \mathbb{R}_+$, and $\lambda_{\mathtt{t}} \in \mathbb{R}_+$, to adjust the weights.

The corresponding optimization problem is

$$\begin{aligned}
\text{minimize} \quad & \mathcal{L} \\
\text{with respect to} \quad & \mathbf{V}_{\mathtt{d}} \in \mathbb{R}^{D_{\mathtt{d}} \times R},\ \mathbf{V}_{\mathtt{t}} \in \mathbb{R}^{D_{\mathtt{t}} \times R},\ \sigma_{\mathtt{e}} \in \mathbb{R}_{++} \\
\text{subject to} \quad & \mathbf{V}_{\mathtt{d}}^{\top}\mathbf{V}_{\mathtt{d}} = \mathbf{I}_R,\ \mathbf{V}_{\mathtt{t}}^{\top}\mathbf{V}_{\mathtt{t}} = \mathbf{I}_R,
\end{aligned}$$

where we assume orthonormality of the embedding dimensions in each domain separately. With this assumption, we prevent the scaling ambiguity caused by heterogeneous objects, and we are able to extract extrapolated information in each dimension of the embedding space. The objective function of the optimization problem is non-convex due to the non-linearity introduced by the Gaussian kernels and global optimization is not possible with known methods. Therefore, we employ a Quasi-Newton method based framework to find a locally optimal solution. In our method, we need to satisfy the orthonormality constraints on the embedding space and the non-negativity constraint on the kernel width.

We can write the gradients of $\mathcal{L}$ with respect to the projection matrices as:

$$\begin{aligned}
\frac{\partial \mathcal{L}}{\partial \boldsymbol{v}_{\mathtt{d},p}} &= 2\frac{\lambda_{\mathtt{c}}}{|\mathcal{I}_{\mathtt{c}}|}\sum_{\mathcal{I}_{\mathtt{c}}} k^i_{\mathtt{c},j}(k^i_{\mathtt{c},j} - s^i_{\mathtt{c},j})\frac{\partial \mathcal{V}^i_{\mathtt{c},j}}{\partial \boldsymbol{v}_{\mathtt{d},p}} + 2\frac{\lambda_{\mathtt{d}}}{|\mathcal{I}_{\mathtt{d}}|}\sum_{\mathcal{I}_{\mathtt{d}}} k^i_{\mathtt{d},j}(k^i_{\mathtt{d},j} - s^i_{\mathtt{d},j})\frac{\partial \mathcal{V}^i_{\mathtt{d},j}}{\partial \boldsymbol{v}_{\mathtt{d},p}} \qquad \forall p \\
\frac{\partial \mathcal{L}}{\partial \boldsymbol{v}_{\mathtt{t},p}} &= 2\frac{\lambda_{\mathtt{c}}}{|\mathcal{I}_{\mathtt{c}}|}\sum_{\mathcal{I}_{\mathtt{c}}} k^i_{\mathtt{c},j}(k^i_{\mathtt{c},j} - s^i_{\mathtt{c},j})\frac{\partial \mathcal{V}^i_{\mathtt{c},j}}{\partial \boldsymbol{v}_{\mathtt{t},p}} + 2\frac{\lambda_{\mathtt{t}}}{|\mathcal{I}_{\mathtt{t}}|}\sum_{\mathcal{I}_{\mathtt{t}}} k^i_{\mathtt{t},j}(k^i_{\mathtt{t},j} - s^i_{\mathtt{t},j})\frac{\partial \mathcal{V}^i_{\mathtt{t},j}}{\partial \boldsymbol{v}_{\mathtt{t},p}} \qquad \forall p,
\end{aligned}$$

where the gradients of the auxiliary variables are found as

$$
\begin{aligned}
\frac{\partial \mathcal{V}^i_{\mathtt{c},j}}{\partial \boldsymbol{v}_{\mathtt{d},p}} &= -\frac{2\boldsymbol{d}_i(\boldsymbol{v}_{\mathtt{d},p}^\top \boldsymbol{d}_i - \boldsymbol{v}_{\mathtt{t},p}^\top \boldsymbol{t}_j)}{\sigma_{\mathtt{e}}^2} && \forall p \\
\frac{\partial \mathcal{V}^i_{\mathtt{d},j}}{\partial \boldsymbol{v}_{\mathtt{d},p}} &= -\frac{2(\boldsymbol{d}_i - \boldsymbol{d}_j)(\boldsymbol{v}_{\mathtt{d},p}^\top \boldsymbol{d}_i - \boldsymbol{v}_{\mathtt{d},p}^\top \boldsymbol{d}_j)}{\sigma_{\mathtt{e}}^2} && \forall p \\
\frac{\partial \mathcal{V}^i_{\mathtt{c},j}}{\partial \boldsymbol{v}_{\mathtt{t},p}} &= -\frac{2\boldsymbol{t}_j(\boldsymbol{v}_{\mathtt{t},p}^\top \boldsymbol{t}_j - \boldsymbol{v}_{\mathtt{d},p}^\top \boldsymbol{d}_i)}{\sigma_{\mathtt{e}}^2} && \forall p \\
\frac{\partial \mathcal{V}^i_{\mathtt{t},j}}{\partial \boldsymbol{v}_{\mathtt{t},p}} &= -\frac{2(\boldsymbol{t}_i - \boldsymbol{t}_j)(\boldsymbol{v}_{\mathtt{t},p}^\top \boldsymbol{t}_i - \boldsymbol{v}_{\mathtt{t},p}^\top \boldsymbol{t}_j)}{\sigma_{\mathtt{e}}^2} && \forall p.
\end{aligned}
$$

Because of the orthonormality constraints, the embedding coordinates of each domain are defined on a Stiefel manifold (i.e., $\mathcal{S}(R, N) = \{\mathbf{E} \in \mathbb{R}^{R \times N} \colon \mathbf{E}\mathbf{E}^\top = \mathbf{I}_R\}$), which is a Riemannian manifold [3]. To satisfy these orthonormality constraints, we need to use a method proved efficient for optimization on Stiefel manifolds to update the embedding coordinates [4].

When learning the kernel width, we apply the *change of variable* technique to work on the logarithmic scale to satisfy the non-negativity constraint. We define a new variable for the logarithm of the kernel width ($\eta_{\mathtt{e}} = \log \sigma_{\mathtt{e}}$) and use an optimization method on this variable. The gradient of $\mathcal{L}$ with respect to $\eta_{\mathtt{e}}$ are

$$
\begin{aligned}
\frac{\partial \mathcal{L}}{\partial \eta_{\mathtt{e}}} = 2\frac{\lambda_{\mathtt{c}}}{|\mathcal{I}_{\mathtt{c}}|} \sum_{\mathcal{I}_{\mathtt{c}}} k^i_{\mathtt{c},j}(k^i_{\mathtt{c},j} - s^i_{\mathtt{c},j}) \frac{\partial \mathcal{V}^i_{\mathtt{c},j}}{\partial \eta_{\mathtt{e}}} \\
+ 2\frac{\lambda_{\mathtt{d}}}{|\mathcal{I}_{\mathtt{d}}|} \sum_{\mathcal{I}_{\mathtt{d}}} k^i_{\mathtt{d},j}(k^i_{\mathtt{d},j} - s^i_{\mathtt{d},j}) \frac{\partial \mathcal{V}^i_{\mathtt{d},j}}{\partial \eta_{\mathtt{e}}} \\
+ 2\frac{\lambda_{\mathtt{t}}}{|\mathcal{I}_{\mathtt{t}}|} \sum_{\mathcal{I}_{\mathtt{t}}} k^i_{\mathtt{t},j}(k^i_{\mathtt{t},j} - s^i_{\mathtt{t},j}) \frac{\partial \mathcal{V}^i_{\mathtt{t},j}}{\partial \eta_{\mathtt{e}}},
\end{aligned}
$$

where the gradients of the auxiliary variables are found as

$$
\begin{aligned}
\frac{\partial \mathcal{V}^i_{\mathtt{c},j}}{\partial \eta_{\mathtt{e}}} &= \frac{2\|\boldsymbol{e}_{\mathtt{d},i} - \boldsymbol{e}_{\mathtt{t},j}\|_2^2}{\sigma_{\mathtt{e}}^2} \\
\frac{\partial \mathcal{V}^i_{\mathtt{d},j}}{\partial \eta_{\mathtt{e}}} &= \frac{2\|\boldsymbol{e}_{\mathtt{d},i} - \boldsymbol{e}_{\mathtt{d},j}\|_2^2}{\sigma_{\mathtt{e}}^2} \\
\frac{\partial \mathcal{V}^i_{\mathtt{t},j}}{\partial \eta_{\mathtt{e}}} &= \frac{2\|\boldsymbol{e}_{\mathtt{t},i} - \boldsymbol{e}_{\mathtt{t},j}\|_2^2}{\sigma_{\mathtt{e}}^2}.
\end{aligned}
$$

Our complete algorithm is an alternating optimization scheme consisting of three main steps:

i. update $\mathbf{V}_{\mathtt{d}}$ given $\mathbf{V}_{\mathtt{t}}$ and σ_e,

ii. update $\mathbf{V}_{\mathtt{t}}$ given $\mathbf{V}_{\mathtt{d}}$ and σ_e, and

iii. update σ_e given $\mathbf{V}_{\mathtt{d}}$ and $\mathbf{V}_{\mathtt{t}}$.

The optimization procedure sequentially updates the decision variables until the stopping criteria are satisfied. Algorithm 1 summarizes the training procedure of our proposed framework.

Algorithm 1 Manifold Optimization based Kernel Preserving Embedding

Input: The scoring functions $s^i_{\mathtt{c},j}$, $s^i_{\mathtt{d},j}$, and $s^i_{\mathtt{t},j}$, the index sets, $\mathcal{I}_{\mathtt{c}}$, $\mathcal{I}_{\mathtt{d}}$, and $\mathcal{I}_{\mathtt{t}}$, subspace dimensionality R, regularization parameters $\lambda_c, \lambda_d, \lambda_t$, relative tolerance parameters ϵ_d, ϵ_t

Initialize $\mathbf{V}_{\mathtt{d}} \in \mathrm{St}(D_d, R), \mathbf{V}_{\mathtt{t}} \in \mathrm{St}(D_t, R)$ randomly with QR factorization, set $\sigma_e = \sqrt{R}$
Calculate the gradients of $\mathcal{L}$ with respect to the projection matrices and logarithm of the kernel width: $\dfrac{\partial \mathcal{L}}{\partial \boldsymbol{v}_{\mathtt{d},p}}, \dfrac{\partial \mathcal{L}}{\partial \boldsymbol{v}_{\mathtt{t},p}}, \dfrac{\partial \mathcal{L}}{\partial \eta_{\mathtt{e}}}$
repeat
  repeat
    update $\mathbf{V}_{\mathtt{d}}$ by using LRBFGS solver
    compute kernel functions $k^i_{\mathtt{c},j}$ and $k^i_{\mathtt{d},j}$
  until stopping criterion is met
  repeat
    update $\mathbf{V}_{\mathtt{t}}$ by using LRBFGS solver
    compute kernel functions $k^i_{\mathtt{c},j}$ and $k^i_{\mathtt{t},j}$
  until stopping criterion is met
  repeat
    update σ_e by using $\dfrac{\partial \mathcal{L}}{\partial \eta_{\mathtt{e}}}$
    compute kernel functions $k^i_{\mathtt{c},j}$, $k^i_{\mathtt{d},j}$ and $k^i_{\mathtt{t},j}$
  until stopping criterion is met
until stopping criterion is met
return $\mathbf{V}_{\mathtt{d}}, \mathbf{V}_{\mathtt{t}}, \sigma_e$

S2 Comparison of MOKPE against the Steepest Descent Method

We compare our algorithm against MKPE [5], a steepest descent based method with Armijo-type line search. We demonstrate the performance of out-of-sample embedding in predicting interactions for unseen drugs. For NR, GPCR, and IC data sets, we apply ten replications of ten-fold cross-validation over drugs to obtain robust results. Since Enzyme data set is much bigger compared to others, achieving convergence within a reasonable timeframe may not be possible for both methods, especially when the subspace dimensionality is larger (i.e., 20 and 25), therefore, it is not included in the comparison. We convert the Matlab implementation of MKPE provided by [5] to R implementation and use it with its default parameters. The code is publicly available at `https://github.com/ocbinatli/mkpe`. We obtain the results of both methods by training them with changing subspace dimensionality parameters taken from $\{5, 10, 15, 20, 25\}$. We use the manifold optimization library `ManifoldOptim` (version `1.0.1`) [6] for employing the algorithm LRBFGS in R programming language (version `4.0.2`) [7]. We set the regularization parameters ($\lambda_{\mathtt{c}}$, $\lambda_{\mathtt{x}}$, $\lambda_{\mathtt{z}}$) to $(1, 0.1, 0.1)$. For MOKPE, we set relative improvement tolerance parameters (ϵ_x, ϵ_z) to $(10^{-3}, 10^{-3})$ as stopping criteria. We use the default values for all other input parameters. We train both of the methods for equal time amount linearly increasing with respect to the subspace dimensionality. Table S1 shows the maximum allowed training time per fold in seconds. All the experiments are run in the same computer cluster family.

Figure S1 gives the average AUROC (area under the receiver operating characteristic curve) values for MKPE and MOKPE. When the subspace dimensionality is smaller than twenty five, we see that MOKPE achieves significantly higher average AUROC values on all the data sets.

Figure S2 shows the objective function value over time for both methods in training data

Table S1: Training Time per fold (in seconds)

	R (subspace dimensionality)				
Data Set	5	10	15	20	25
NR	10	20	30	40	50
GPCR	300	600	900	1200	1500
IC	400	800	1200	1600	2000

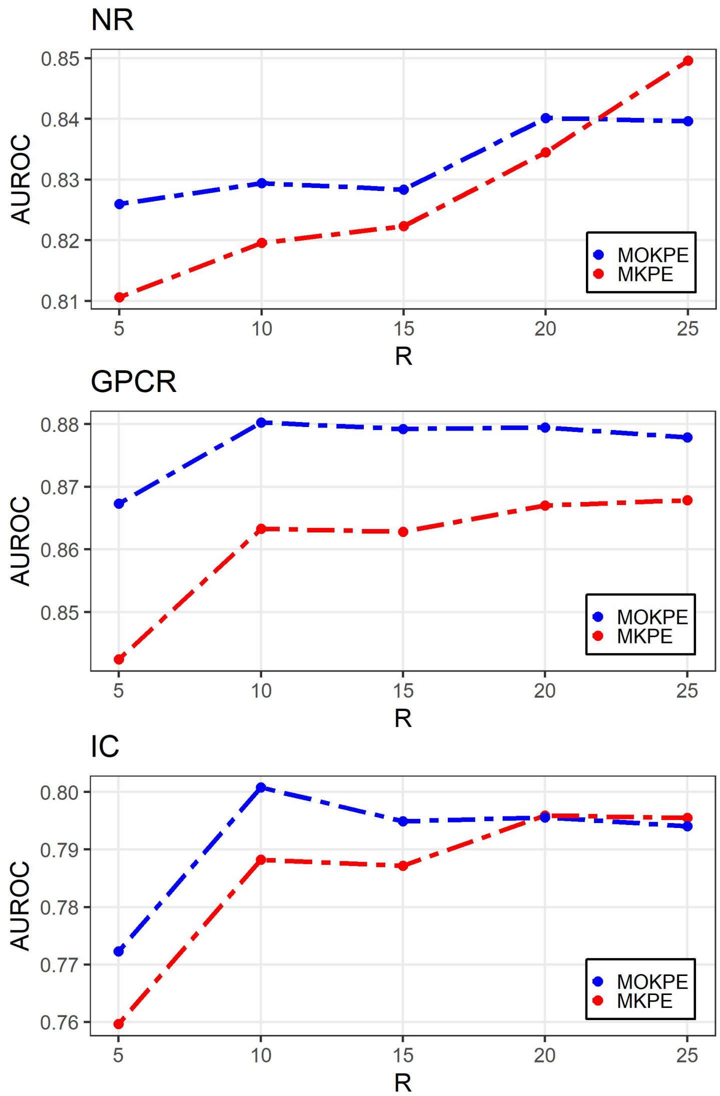

Figure S1: The prediction performances of MKPE and MOKPE with changing subspace dimensionality on the NR, GPCR and IC data sets in terms of average AUROC values.

sets. Each smooth curve is a LOESS (locally estimated scatterplot smoothing) fitted curve. MKPE starts better but more likely to "get stuck in a local optima" later on. Also, the iterations are less costly time-wise for MKPE, which provides an advantage over MOKPE at the starting phase.

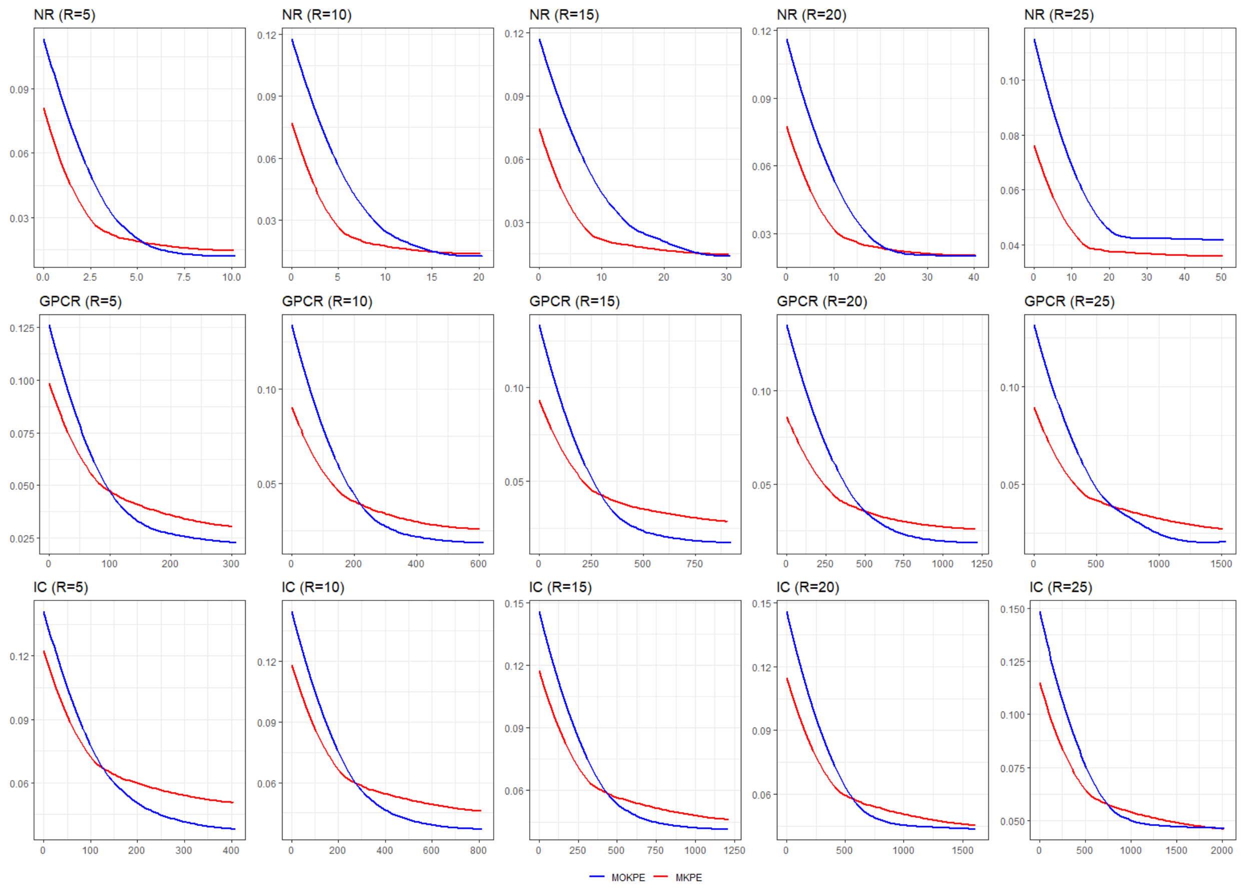

Figure S2: Objective function values vs. processing time (in seconds) for the training sets on MKPE and MOKPE with changing subspace dimensionality on the NR, GPCR and IC data sets.

S3 Parameter Settings

When running the compared methods, we used the reported best hyper-parameters from the sources [8, 9, 10]. The hyper-parameters are as follows:

- <u>LapRLS</u>: $\beta_d = 0.3, \beta_p = 0.3, \gamma_{d1} = 1, \gamma_{p1} = 1, \gamma_{d2} = 0.01, \gamma_{p2} = 0.01$
- <u>DLapRLS</u>: NR data set: regularization terms $\lambda_1 = 0.9$, $\lambda_2 = 0.9$, number of iterations $= 10$. GPCR data set: $\lambda_1 = 0.95$, $\lambda_2 = 0.9$, number of iterations $= 10$. IC data set: $\lambda_1 = 0.9$, $\lambda_2 = 1$, number of iterations $= 5$. Enzyme data set: $\lambda_1 = 0.9$, $\lambda_2 = 0.9$, number of iterations $= 5$.
- <u>RLS-WNN</u>: NR data set: regularization term $\sigma = 0.5$, weight $\alpha = 0.5$, kernel bandwidth $\eta = 0.6$. GPCR data set: $\sigma = 0.5, \alpha = 1, \eta = 0.7$. IC data set: $\sigma = 0.25, \alpha = 1, \eta = 0.7$. Enzyme data set: $\sigma = 0.5, \alpha = 1$, $\eta = 0.7$.
- <u>KBMF2K</u>: NR data set: subspace dimensionality $R = 50$. GPCR data set: $R = 100$. IC data set: $R = 100$. Enzyme data set: $R = 200$.
- <u>CMF</u>: number of iterations $= 2$, dimensionality of the feature space $k = 100$. NR data set: weight parameters $\lambda_l = 1, \lambda_d = 4, \lambda_t = 32$. GPCR data set: $\lambda_l = 2, \lambda_d = 8, \lambda_t = 0.125$. IC data set: $\lambda_l = 4, \lambda_d = 32, \lambda_t = 0.25$. Enzyme data set: $\lambda_l = 2, \lambda_d = 64, \lambda_t = 0.125$.
- <u>WGRMF</u>: number of iterations $= 2$, $k = 100$. NR data set: regularization terms $\lambda_l = 0.0625$, $\lambda_d = 0.05$, $\lambda_t = 0.1$, number of neighbors $p = 2$. GPCR data set: $\lambda_l = 0.0625$,

$\lambda_d = 0.05$, $\lambda_t = 0.01, p = 7$. IC dataset: $\lambda_l = 0.25$, $\lambda_d = 0.15$, $\lambda_t = 0.01$, $p = 6$. Enzyme dataset: $\lambda_l = 0.25$, $\lambda_d = 0.2$, $\lambda_t = 0.2$, $p = 4$.

- GRGMF: number of iterations = 100, the dimension of the subspace expanded by self-representing vectors $K = 150$, number of neighbors $k = 5$, decay term $\eta = 0.5$, matrix regularization parameters $\lambda = 0.0333, \beta = 4$, graph regularization parameters $r_1 = 0.5$, $r_2 = 1$, the weight assigned to known link $c = 5$.

- HGBI: the decay factor $\alpha = 0.5$.

References

[1] Schölkopf B, Tsuda K, Vert JP. Kernel methods in computational biology. MIT press; 2004.

[2] Schölkopf B, Smola AJ, Bach F, et al. Learning with kernels: support vector machines, regularization, optimization, and beyond. MIT press; 2002.

[3] Chakraborty R, Vemuri BC. Statistics on the Stiefel manifold: Theory and applications. The Annals of Statistics. 2019;47(1):415–438.

[4] Boumal N. An introduction to optimization on smooth manifolds. Cambridge University Press; 2023. Available from: `https://www.nicolasboumal.net/book`.

[5] Gönen M. Embedding heterogeneous data by preserving multiple kernels. In: 21st European Conference on Artificial Intelligence, ECAI 2014. IOS Press; 2014. p. 381–386.

[6] Martin S, Raim AM, Huang W, Adragni KP. ManifoldOptim: An R Interface to the ROPTLIB Library for Riemannian Manifold Optimization. Journal of Statistical Software. 2020;93:1–32.

[7] R Core Team. R: A Language and Environment for Statistical Computing. Vienna, Austria; 2021. Available from: `https://www.R-project.org/`.

[8] Ezzat A, Wu M, Li XL, Kwoh CK. Computational prediction of drug–target interactions using chemogenomic approaches: an empirical survey. Briefings in bioinformatics. 2019;20(4):1337–1357.

[9] Ding Y, Tang J, Guo F. Identification of drug–target interactions via dual laplacian regularized least squares with multiple kernel fusion. Knowledge-Based Systems. 2020;204:106254.

[10] Zhang ZC, Zhang XF, Wu M, Ou-Yang L, Zhao XM, Li XL. A graph regularized generalized matrix factorization model for predicting links in biomedical bipartite networks. Bioinformatics. 2020;36(11):3474–3481.
